# High low-density lipoprotein cholesterol level is associated with an increased risk of incident early-onset vasomotor symptoms

**DOI:** 10.1038/s41598-022-19028-4

**Published:** 2022-08-27

**Authors:** Hye Rin Choi, Yoosoo Chang, Yejin Kim, Jeonggyu Kang, Min-Jung Kwon, Ria Kwon, Ga-Young Lim, Kye-Hyun Kim, Hoon Kim, Yun Soo Hong, Di Zhao, Juhee Cho, Eliseo Guallar, Hyun-Young Park, Seungho Ryu

**Affiliations:** 1 Center for Cohort Studies, Total Healthcare Center, Kangbuk Samsung Hospital, Sungkyunkwan University School of Medicine, Seoul, 04514 Republic of Korea; 2 Institute of Medical Research, Sungkyunkwan University School of Medicine, Suwon, 16419 Republic of Korea; 3 Department of Occupational and Environmental Medicine, Kangbuk Samsung Hospital, Sungkyunkwan University School of Medicine, Seoul, 03181 Republic of Korea; 4 Department of Clinical Research Design & Evaluation, Samsung Advanced Institute for Health Sciences & Technology, Sungkyunkwan University, Seoul, 06355 Republic of Korea; 5 Department of Laboratory Medicine, Kangbuk Samsung Hospital, Sungkyunkwan University School of Medicine, Seoul, 04514 Republic of Korea; 6 Department of Obstetrics and Gynecology, Kangbuk Samsung Hospital, Sungkyunkwan University School of Medicine, Seoul, 03181 Republic of Korea; 7 Department of Obstetrics and Gynecology, Seoul National University College of Medicine, Seoul, 03080 Republic of Korea; 8 Departments of Epidemiology and Medicine, and Welch Center for Prevention, Epidemiology, and Clinical Research, Johns Hopkins University Bloomberg School of Public Health, Baltimore, MD 21205 USA; 9 Department of Precision Medicine, National Institute of Health, Korea Disease Control and Prevention Agency, Cheongju, 28159 Republic of Korea

**Keywords:** Risk factors, Lifestyle modification, Preventive medicine, Geriatrics, Epidemiology

## Abstract

We investigated the associations between serum lipid profiles and risk of early-onset vasomotor symptoms (VMSs) in premenopausal women. This cohort study comprised 2,540 premenopausal women aged 42–52 years without VMSs at baseline (median follow-up: 4.4 years). VMSs, including hot flashes and night sweats, were assessed using the Menopause-Specific Quality of Life questionnaire (Korean version). Early-onset VMSs were defined as VMSs that occurred premenopause; moderate/severe VMSs were defined as a score of ≥ 3 points (range: 0 to 6, 6 being most bothersome). Cox proportional hazard regression models were used to estimate hazard ratios with 95% confidence intervals (CI) for the development of VMSs across the lipid levels. Higher low-density lipoprotein (LDL) cholesterol levels were positively associated with increased risk of early-onset VMSs. Compared to the < 100 mg/dL LDL group, the multivariable-adjusted hazard ratios (HRs) with 95% confidence intervals (CIs) for incident VMSs were 1.19 (1.03–1.37) and 1.20 (1.03–1.40) in participants with LDL cholesterol levels of 100–129 mg/dL and ≥ 130 mg/dL, respectively (*P* for trend = 0.027). The multivariable-adjusted HR for incident moderate/severe VMSs was 1.37 (95% CI: 1.08–1.73) in participants with LDL ≥ 130 mg/dL, compared to those with LDL < 100 mg/dL. Meanwhile, triglycerides and total and high-density lipoprotein cholesterol levels were not significantly associated with early-onset VMSs risk in premenopausal women. Premenopausal women with high serum LDL cholesterol concentrations had a higher risk of incident early-onset VMSs. Further studies should confirm our findings and examine whether LDL-lowering interventions reduce the risk of early-onset VMSs among women during menopause transition.

## Introduction

Menopause is characterized by physiological and psychological changes in a woman’s life^1^. Vasomotor symptoms (VMSs), including hot flashes and night sweats, are considered the predominant symptoms of menopause^2^. Approximately 60–80% of middle-aged women experience some degree of VMSs during menopausal transition^3^. VMSs can occur near the final menstrual period and last for approximately one year after menopause; however, recent studies show that VMSs can start far earlier than previously reported, even during the premenopausal and early menopausal transition stages and can persist longer than 10 years after the final menopausal period (FMP)^4,5^. The severity and duration of VMSs vary among individuals. Frequent, severe, and long-lasting VMSs have been associated with adverse cardiovascular disease (CVD) risk factors, such as high blood pressure, increased intima-media thickness, obesity, endothelial dysfunction, and adverse cholesterol levels^6–9^. However, the pathophysiological mechanisms that explain the association between CVD risk profiles and VMSs remain unclear.

Among the CVD risk factors, abnormal lipid profiles such as high total and low-density lipoprotein (LDL) cholesterol and triglyceride (TG) levels and low high-density lipoprotein (HDL) cholesterol levels are the most common abnormalities in postmenopausal women because hormonal changes affect blood cholesterol concentrations^10,11^. During menopausal transition, endogenous estradiol (E2) levels decline, increasing the risk of CVD in women after the age of 50 years^12–14^. The timing of declining E2 in the menopausal transition has been related to the incident timing of VMSs, and women who experience more frequent VMSs may also have significantly lower E2 concentrations^12,15^. Furthermore, elevated LDL cholesterol, a major risk factor for CVD, has been associated with endothelial dysfunction^16,17^. Recent studies have suggested that abnormal lipid profiles, especially LDL cholesterol, may be associated with the risk of VMSs during menopausal transition; however, the previous results were inconsistent, and the relationship were not well elucidated. Therefore, we aimed to examine whether adverse lipid profiles are associated with an increased risk of early-onset VMSs in premenopausal women. Furthermore, we investigated which lipid factors most affect the risk of early-onset and severe VMSs.

## Results

Table 1 shows the characteristics of 2,540 VMS-free premenopausal women at baseline. During the follow-up period, a total of 1,243 women (48.9%) developed early-onset VMSs. Overall, the mean age was 44.6 ± 2.3 years. The mean BMI, alcohol intake, and age at menarche were 22.3 ± 3.0, 4.4 ± 7.1, and 13.9 ± 1.4, respectively. The majority of women (89.5%) had one or more parities, and 279 (11.0%) were ever-smokers. Compared with women who did not develop VMSs, women who developed VMSs were likely to have higher levels of total cholesterol, LDL cholesterol, triglycerides, and lower HDL levels, but these differences did not reach statistical significance for total and LDL cholesterols.

**Table 1:** Demograpic and clinical characteristics of study participants.

Characteristics	Overall (n = 2,540)	Without new early-onset VMSs (n = 1,297)	With new early-onset VMSs (n = 1,243)	p-value
Age (y)	44.6 ± 2.3	44.4 ± 2.3	44.9 ± 2.4	< 0.001
BMI (kg/m^2^)	22.3 ± 3.0	22.1 ± 3.0	22.5 ± 2.8	< 0.001
Waist circumference (cm)	75.5 ± 7.6	74.9 ± 7.7	76.2 ± 7.5	< 0.001
Age at menarche (years)	13.91 ± 1.4	13.9 ± 1.4	13.97 ± 1.4	0.044
**Parity**				
No child	257 (10.6)	146 (11.7)	111 (9.3)	0.032
1 child	605 (24.9)	320 (25.7)	285 (24.1)	
2 children	1393 (57.3)	702 (56.4)	691 (58.1)	
≥ 3 or more children	178 (7.3)	77 (6.2)	101 (8.5)	
Ever smoker	279 (11.0)	146 (11.3)	133 (10.7)	0.400
Alcohol intake (g/day)	4.4 ± 7.1	4.28 ± 6.4	4.5 ± 7.9	0.396
< 10 g/day drinker	2133 (84.0)	1089 (84.0)	1044 (84.0)	0.043
10–40 g/day drinkers	253 (10.0)	134 (10.3)	119 (9.6)	
> 40 g/day drinkers	20 (0.8)	4 (0.3)	16 (1.3)	
**HEPA**				
Low	1287 (51.0)	688 (53.4)	599 (48.5)	< 0.001
Moderate	854 (33.9)	439 (34.2)	415 (33.6)	
High	382 (15.1)	160 (12.4)	222 (18.0)	
High education^†^	2042 (81.8)	1067 (84.2)	973 (79.2)	0.001
Hypertension	86 (3.4)	43 (3.3)	43 (3.5)	0.846
Diabetes	35 (1.4)	18 (1.4)	17 (1.4)	0.962
Systolic BP (mmHg)	103.0 ± 11.0	102.4 ± 10.7	103.8 ± 11.3	0.001
Diastolic BP (mmHg)	66.2 ± 8.6	65.9 ± 8.5	66.5 ± 8.7	0.059
Glucose (mg/dL)	92.5 ± 10.8	92.1 ± 10.6	92.9 ± 11.3	0.055
HOMA-IR†	1.1 [0.7, 1.6]	1.1 [0.7, 1.5]	1.1 [0.8–1.6]	0.135
hsCRP (mg/L) ^‡^	0.3 [0.2, 0.6]	0.3 [0.2, 0.5]	0.3 [0.2, 0.6]	0.335
Total cholesterol (mg/dL)	191.5 ± 30.3	191.1 ± 30.8	191.8 ± 29.7	0.572
< 200	1594 (62.8)	816 (62.9)	778 (62.6)	0.972
200– < 240	771 (30.3)	391 (30.1)	380 (30.6)	
≥ 240	175 (6.9)	90 (6.9)	85 (6.8)	
LDL-C (mg/dL)	118.2 ± 28.2	117.8 ± 28.4	118.5 ± 28.0	0.526
< 100	676 (26.6)	365 (28.2)	311 (25.0)	0.202
100– < 130	1038 (40.9)	517 (39.8)	521 (42.0)	
≥ 130	826 (32.5)	415 (32.0)	411 (33.0)	
HDL-C (mg/dL)	67.4 ± 15.9	68.0 ± 16.0	66.7 ± 15.8	0.037
< 50 (abnormal)	301 (11.9)	141 (10.9)	160 (12.9)	0.210
50– < 60	551 (21.7)	276 (21.3)	275 (22.1)	
≥ 60	1688 (66.4)	880 (67.8)	808 (64.8)	
Non-HDL (mg/dL)	124.1 ± 30.8	123.1 ± 30.7	125.1 ± 30.8	0.102
< 130	1521 (59.8)	788 (60.7)	733 (58.9)	0.574
130– < 160	697 (27.5)	352 (27.2)	345 (27.7)	
≥ 160	322 (12.7)	157 (12.1)	165 (13.3)	
Triglycerides (mg/dL)	73 [56, 97]	71 [55, 95]	75 [58, 100]	0.006
< 100	1939 (76.3)	1014 (78.1)	925 (74.4)	0.059
100– < 150	445 (17.5)	214 (16.5)	231 (18.6)	
≥ 150	156 (6.2)	69 (5.4)	87 (7.1)	
Apo A-1(mg/dL)*	155.8 ± 23.9	155.9 ± 23.2	155.7 ± 24.7	0.896
< 140 (abnormal)	449 (26.0)	224 (25.1)	225 (27.0)	0.354
≥ 140	1276 (74.0)	669 (74.9)	607 (73.0)	
ApoB (mg/dL)*	89.6 ± 21.1	89.1 ± 20.8	90.1 ± 21.5	0.345
< 90	929 (53.9)	480 (53.8)	449 (54.0)	0.929
≥ 90 (abnormal)	796 (46.1)	413 (46.3)	383 (46.0)	
Apo B/Apo A-1 ratio	0.6 ± 0.2	0.6 ± 0.2	0.6 ± 0.2	0.260

Data presented are mean ± standard deviation, median (interquartile range), or percentage.

VMSs, vasomotor symptoms; LDL, low-density lipoprotein; HDL, high-density lipoprotein; Apo A-1, apolipoprotein A-1; ApoB, apolipoprotein B; BMI, body mass index; HEPA, health-enhancing physical activity; BP, blood pressure; HOMA-IR, homeostatic model assessment of insulin resistance; hsCRP, high-sensitivity C-reactive protein.

* among 1,725 participants.

^†^ ≥ college graduate.

Longitudinal associations between lipid profiles and the incidence of early-onset VMSs among premenopausal women free of VMSs at baseline are presented in Table 2. During 11,196.18 person-years of follow-up, 1,243 cases of incident early-onset VMSs were found (incidence rate, 11.1 per 100 person-years). Median follow-up duration was 4.4 years (interquartile range, 3.4–5.6 years). Higher LDL cholesterol concentrations were significantly associated with an increased risk of incident early-onset VMSs. The age-adjusted HRs (95% CIs) for incident early-onset VMSs were 1.17 (1.02–1.35) in people with LDL levels of 100–129 mg/dL and 1.23 (1.06–1.42) in women with LDL levels ≥ 130 mg/dL, compared with women with LDL levels < 100 mg/dL (*P* for trend = 0.008). After adjustments for age, BMI, SBP, DBP, diabetes, education levels, parity, physical activity, smoking status, and alcohol intake, the HRs (95% CIs) for incident early-onset VMSs were 1.19 (1.03–1.37) and 1.20 (1.03–1.40) for of the 100 ≤ LDL < 130 mg/dL and > 130 mg/dL groups, respectively (*P* for trend = 0.027; and also see Supplementary Figure 1). However, other lipid profiles, including total cholesterol, HDL cholesterol, and TG, were not significantly associated with incident VMSs during the follow-up period before and after adjusting for potential confounders. Furthermore, in the analyses using separately hot flushes and night sweats as the outcome, we also found that higher LDL cholesterols were significantly associated with each outcome (Supplementary Table 1). On the other hand, there was no significant relationship of other lipids with either hot flushes or night sweats.

**Table 2:** Longitudinal association between lipid profiles and incidence of VMSs among premenopausal women free of VMSs at baseline (n = 2,540)*.

Lipid profiles	Person-years (PY)	Early-onset VMSs	Incidence rate (cases per 100 PY)	Age-adjusted HR (95% CI)	Multivariable-adjusted HR (95% CI)
**Total cholesterol (mg/dL)**					
< 200	7,083.30	778	11.0	Reference	Reference
200– < 240	3,363.14	380	11.3	1.03 (0.91–1.16)	0.99 (0.87–1.12)
≥ 240	749.75	85	11.3	0.96 (0.77–1.20)	0.92 (0.73–1.15)
*P for trend*				0.976	0.534
**LDL-C (mg/dL)**					
< 100	3091.91	311	10.1	Reference	Reference
100– < 130	4,570.21	522	11.4	1.17 (1.02–1.35)	1.19 (1.03–1.37)
≥ 130	3,534.06	411	11.6	1.23 (1.06–1.42)	1.20 (1.03–1.40)
*P for trend*				0.008	0.027
**HDL-C (mg/dL)**					
< 50 (abnormal)	1366.18	160	11.7	0.97 (0.82–1.15)	0.93 (0.77–1.11)
50– < 60	2,441.16	275	11.3	0.97 (0.84–1.11)	0.96 (0.83–1.10)
≥ 60	7,388.84	808	10.9	Reference	Reference
*P for trend*				0.629	0.366
**Non-HDL (mg/dL)**					
< 130	6767.28	733	10.8	Reference	Reference
130– < 160	3023.7	345	11.4	1.00 (0.88–1.13)	0.97 (0.85–1.11)
≥ 160	1405.2	165	11.7	1.03 (0.87–1.23)	0.98 (0.82–1.17)
*P for trend*				0.765	0.759
**Triglycerides (mg/dL)**					
< 100	8605.29	925	10.7	Reference	Reference
100– < 150	1921.46	231	12.0	1.13 (0.98–1.31)	1.09 (0.93–1.27)
≥ 150	669.43	87	13.0	1.18 (0.94–1.47)	1.06 (0.83–1.35)
*P for trend*				0.041	0.359

VMSs, vasomotor symptoms; HR, hazard ratio; *CI*, confidence interval; LDL, low-density lipoprotein; HDL, high-density lipoprotein.

*The multivariable model was adjusted for age, body mass index, systolic and diastolic blood pressure, diabetes, educational level, parity, physical activity, smoking status, and alcohol intake.

In the sensitivity analysis for incident moderate/severe VMSs as an endpoint (Table 3), LDL cholesterol was significantly associated with an increased risk of moderate/severe VMSs in premenopausal women. The multivariable-adjusted HRs (95% CI) for developing moderate/severe VMSs were 1.35 (1.08–1.67) in women with LDL levels of 100–129 mg/dL and 1.37 (1.08–1.73) in women with LDL levels ≥ 130 mg/dL (*P* for trend = 0.013) when compared with the reference group (LDL < 100 mg/dL). Other lipids, including TG, total cholesterol, and HDL cholesterol levels, were not significantly associated with incident moderate/severe VMSs in this population. In the additional sensitivity analyses including women with TG > 400 mg/dl (n = 3), the results were almost identical to those in Tables 2 and 3 (Supplementary Tables 2 and 3).

**Table 3:** Longitudinal association between lipid profiles and incidence of moderate/severe VMSs among premenopausal women free of VMSs at baseline (n = 2,540)*.

Lipid profiles	Person-years (PY)	Onset of severe VMSs	Incidence rate (cases per 100 PY)	Age-adjusted HR (95% CI)	Multivariable-adjusted* HR (95% CI)
**Total cholesterol (mg/dL)**					
< 200	7,426.32	342	4.6	Reference	Reference
200– < 240	3,535.44	183	5.2	1.12 (0.94–1.34)	1.06 (0.88–1.27)
≥ 240	781.69	44	5.6	1.13 (0.82–1.54)	1.08 (0.78–1.48)
*P for trend*				0.212	0.508
**LDL-C (mg/dL)**					
< 100	3239.99	121	3.7	Reference	Reference
100– < 130	4,791.83	246	5.1	1.37 (1.10–1.70)	1.35 (1.08–1.67)
≥ 130	3,711.63	202	5.4	1.46 (1.16–1.83)	1.37 (1.08–1.73)
*P for trend*				0.002	0.013
**HDL-C (mg/dL)**					
< 50 (abnormal)	1,436.57	82	5.7	1.06 (0.83–1.35)	0.97 (0.75–1.26)
50– < 60	2,555.09	129	5.0	1.02 (0.84–1.25)	0.98 (0.80–1.21)
≥ .2	7751.79	358	4.6	Reference	Reference
*P for trend*				0.616	0.793
**Non-HDL (mg/dL)**					
< 130	7089.24	320	4.5	Reference	Reference
130– < 160	3187.54	163	5.1	1.07 (0.88–1.29)	1.00 (0.82–1.22)
≥ 160	1466.67	86	5.9	1.21 (0.95–1.54)	1.12 (0.87–1.43)
*P for trend*				0.120	0.472
**Triglycerides (mg/dL)**					
< 100	9008.44	415	4.6	Reference	Reference
100– < 150	2032.70	110	5.4	1.15 (0.93–1.41)	1.05 (0.84–1.31)
≥ 150	702.31	44	6.3	1.27 (0.93–1.73)	1.07 (0.76–1.50)
*P for trend*				0.069	0.608

Abbreviations: VMSs, vasomotor symptoms; HR, hazard ratio; *CI*, confidence interval; LDL, low-density lipoprotein; HDL, high-density lipoprotein.

*The multivariable model was adjusted for age, body mass index, systolic and diastolic blood pressure, diabetes, educational level, parity, physical activity, smoking status, and alcohol intake.

## Discussions

In this cohort study of premenopausal women, LDL cholesterol concentrations showed a dose–response relationship with an increased risk of early-onset VMSs, and this association remained significant after adjustments for age, BMI, BP, diabetes, education status, parity, physical activity, smoking status, and alcohol intake. Increased LDL levels were also significantly associated with the risk of moderate/severe VMSs, and this association was stronger than that in the analysis without consideration of VMSs severity. Conversely, other lipid profiles, such as TG, total cholesterol, and HDL cholesterol levels, were not significantly associated with the risk of either overall VMSs or moderate/severe VMSs.

Several previous epidemiological studies have investigated the relationship between lipids and VMSs with conflicting results^8,12,15,18^. In a large population-based cross-sectional study that examined the associations between prevalent VMSs and CVD risk factors in 5,857 postmenopausal women without CVD at baseline, women with VMSs had unfavorable CVD risk profiles, such as abnormal lipid levels, high BP, and high BMI^15^. On the other hand, another cross-sectional study of recently postmenopausal women showed no differences in lipids, lipoproteins, sex hormone binding globulin, or high-sensitive C-reactive protein between women with and without of mild hot flashes^18^. However, these findings are not directly comparable to ours due to the cross-sectional design, relatively higher age ranges and distinct racial compositions of the populations in the studies.

A longitudinal analysis from the Study of Women’s Health Across the Nation (SWAN) suggested that frequent VMSs were associated with increased levels of LDL and HDL cholesterol, triglycerides, apolipoprotein A-1, and apolipoprotein B during an eight-year follow-up period, suggesting a close link between VMSs and adverse lipid profiles^8^. A recent ancillary study of 522 middle-aged women from the SWAN, which assessed lipoprotein particles and size, also indicated that frequent VMSs was significantly associated with higher concentration of LDL cholesterols and intermediate LDL particles^12^. Our study demonstrated that abnormal LDL cholesterol among lipid profiles was significantly associated with increased risk of early onset VMSs, including both hot flashes and night sweats. However, since lipid particle and size were not measured in detail, we could not further evaluate the various relationships between lipid profiles and early onset VMSs according to lipid particles and size. Moreover, existing studies regarding the risk factors of VMSs have largely been confined to Caucasian populations. Our data on homogenously Asian female population, which is likely to have different genetic and environmental liabilities from other ethnic/racial groups, add to the previous work. The early-occurring VMSs may have important implications in women’s cardiovascular health, as timing of the VMSs onset may have substantial impact on CVD-related prognosis^19^. Women with early-onset VMSs had poorer endothelial function, which was characterized by the higher intima media thickness, a well-known subclinical CVD marker for future CVD event, as well as higher CVD mortality compared to those with consistently low VMSs throughout menopausal transition^19,20^. Although other cholesterols did not show significant relationship with VMSs, we carefully suggested that elevated LDL levels may serve as an indicator that identifies premenopausal women at high risk of VMSs. It might allow for early intervention to reduce the risk of VMSs and the future CVD events. Furthermore, a previous study of 17,473 postmenopausal women demonstrated that low-fat diets with high fruit, vegetable, and whole grain consumptions were associated with reduced risk of VMS. Women who lost weight as a result of the intervention had reduced or eliminated VMSs, compared to women who maintained their body weight^21^. Another previous review paper investigated whether lifestyle factors including smoking habits could modify vasomotor symptoms. These results showed that smokers had significantly higher risks of having VMSs than non-smokers, suggesting that smoking cessation may reduce severity of VMSs in women^22^.

The mechanism underlying the association between increased LDL cholesterol and risk of VMSs is poorly understood. According to the recent findings, VMSs, including hot flashes and night sweats, in middle-aged women are reflective of CVD risk and are associated with various markers of endothelial dysfunction^6,19,20^. Endothelial dysfunction is considered a major initiating event in the development of atherosclerosis and subsequent CVD development^23^, and there is evidence that chronic elevation of plasma LDL cholesterol is a major determinant of endothelial dysfunction^17^. A previous cross-sectional study using ambulatory hot flash monitoring, physiologically assessed hot flashes were associated with low flow-mediated dilation among relatively young women aged 40–53 years, while the same associations were not observed in older women aged 54–60 years, suggesting that VMSs experienced at an earlier age may be more strongly associated with vascular dysfunction^24^. Taken together, elevated LDL cholesterol among abnormal cholesterol status may be an early sign of adverse changes in vascular status which could later manifest as VMSs. Abnormal cholesterol profile, high LDL cholesterol in particular, may also cause changes in autonomic nervous system balance favoring increased sympathetic and decreased parasympathetic tone, which is one of the proposed mechanisms of VMSs pathogenesis^23,25^.

Previous studies have suggested that estrogen deficit accompanying menopause may play an important role in worsening CVD risk profiles as well as VMSs during menopausal transition^26^. However, based on several reports, the association between CVD risk factors and VMSs does not seem to be not fully explained by E2 levels^8,15^. However, in a study by Thurston et al., the association between LDL cholesterol and VMSs was attenuated after controlling for follicle-stimulating hormone (FSH), whereas the association with other lipid parameters remained unaffected^8^. Whether this association would also be true of our study could not be determined as we did not have information on FSH or E2. Our population was composed exclusively of premenopausal women, who had not reached menopause, and presumably, the effect of E2 or FSH status in premenopausal stage might not be as substantial as in those in later stages of menopausal transition or post-menopause. Nevertheless, additional investigations are warranted to elucidate mechanisms linking LDL cholesterol and early-onset VMSs and whether reproductive hormone status plays a role in the association.

There are some limitations to our study. First, specific measures of reproductive hormones including estrogen or FSH, which may affect the association between lipids and VMSs, were not available for our study. Further studies are needed to determine the relationship between estrogen levels in the association between lipid profiles and VMSs. Second, confounders including smoking status, alcohol intake, and physical activity were measured using self-reported questionnaires, which may lead to misclassification. Moreover, there remains a possibility of residual confounding due to unmeasured confounders. Finally, our study cohort was composed of middle-aged Korean women. Thus, our results may not be generalizable to other populations of different races or ethnic compositions. Nonetheless, our study suggests that a high LDL cholesterol level might be an independent risk factor for early-onset VMSs based on the longitudinal cohort data. We also evaluated the bothersome degree of VMSs using questionnaires and presented a significant association of LDL levels with incident moderate/severe VMSs. Furthermore, our study included a prospective design, a large sample size of a well-characterized population of premenopausal women, and the use of carefully standardized clinical, lifestyle, and laboratory measures, which allowed us to account for multiple potential confounders.

In conclusion, high LDL cholesterol levels were significantly associated with an increased risk of early-onset VMSs in premenopausal Korean women. Moreover, women with hyper-LDL-cholesterolemia were likely to develop moderate/severe VMSs. Further studies are required to clarify the independent association of LDL cholesterol with early-onset VMSs considering estrogen and other sex hormone effects in women before menopause.

## Methods

### Study population

**Figure 1:**
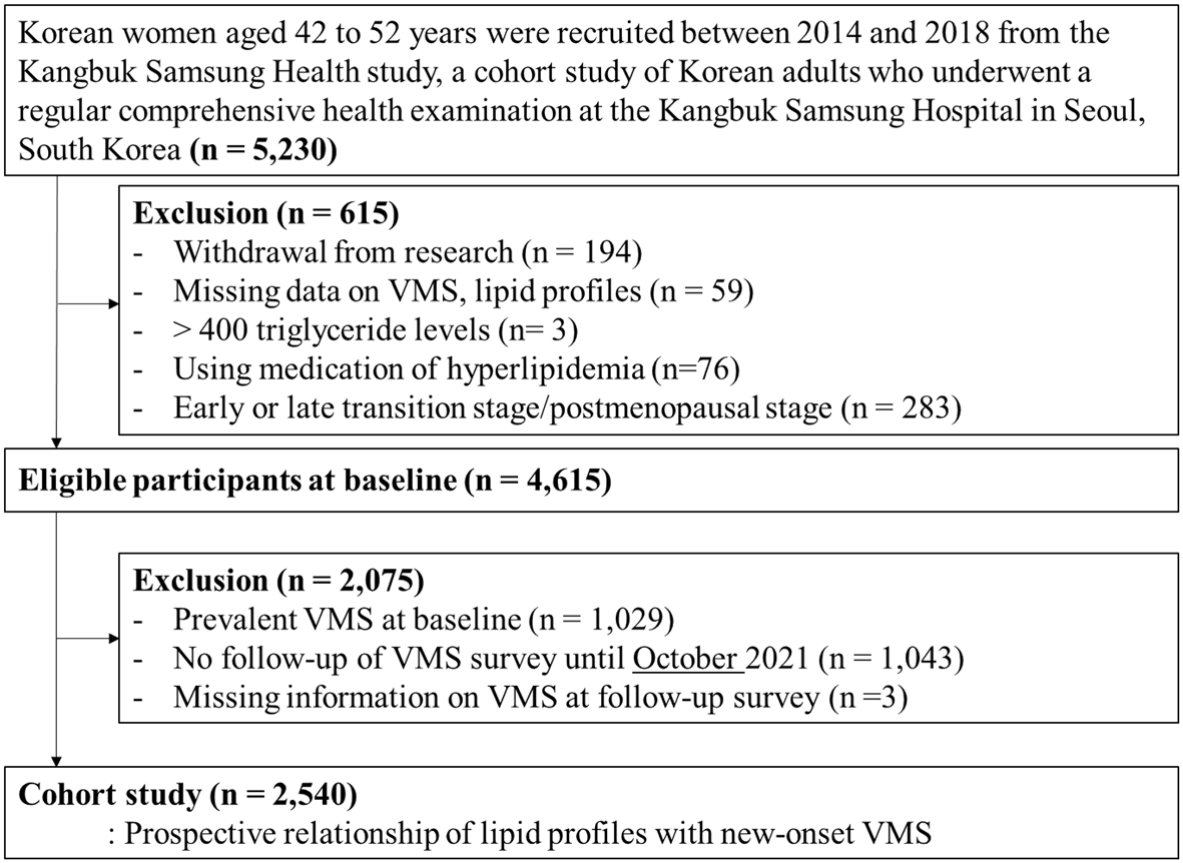
Flow diagram for the selection of the study population.

In this longitudinal study of middle-aged Korean women, we recruited participants between 2014 and 2018 from the Kangbuk Samsung Health Study, a cohort study of Korean men and women who underwent annual or biannual comprehensive health examinations at Kangbuk Samsung Hospital Total Healthcare Center clinics in Seoul and Suwon, South Korea. The eligibility criteria for enrollment in this longitudinal study included: (1) age 42–52 years; (2) no history of hysterectomy, oophorectomy, or hormone replacement therapy; (3) at least one menstrual period in the three months prior to the health screening check-up and no amenorrhea lasting for ≥ 60 days; and 4) no history of a chronic disease that may affect menstrual cycles (malignancy, renal failure, and hypo- or hyperthyroidism). Among the 5,230 women initially enrolled, 283 women in the early or late menopausal transition or postmenopausal stages were excluded because we only included women in the premenopausal stage in this longitudinal study. We also excluded participants who withdrew (n = 194), had no information on VMSs or lipid profiles (n = 59), used lipid-lowering drugs (n = 76), and had serum TG levels > 400 mg/dL (n = 3). In order to investigate longitudinal associations, women who had VMSs at baseline (n = 1,029), did not receive follow-up examinations (n = 1,043), and had missing information on VMSs (n = 3) during the follow-up period were excluded (Fig. 1). Therefore, 2,540 participants were ultimately included in this study.

This study was approved by the Institutional Review Board of Kangbuk Samsung Hospital (IRB No. KBSMC 2022–01-014). All methods in this cohort study were performed in accordance with relevant guidelines and regulations. All study participants provided written informed consent.

### Measurements

Information regarding demographic characteristics, health-related behaviors, medication usage, and reproductive factors was obtained using a standardized, structured, and self-administered questionnaire. Regarding health-related lifestyle factors, smoking status was categorized as never smokers and ever-smokers. Because the proportion of former/current smokers was very low in women, we did not differentiate former from current smokers. Never smokers were defined as women who had smoked less than 100 cigarettes in their lifetime and ever-smokers were defined as women who had smoked equal to or more than 100 cigarettes during their lifetime^6^. Levels of alcohol intake were categorized as < 10 g/day, 10–40 g/day, and > 40 g/day. Physical activity was assessed using the validated Korean version of the International Physical Activity Questionnaire short form and was divided into three groups: inactive, minimally active, and health-enhancing physical activity^27,28^. Education status was dichotomized as less than university graduates and equal to or greater than university graduates.

Reproductive factors included age at menarche, parity, and menopausal status. Parity, defined as the number of pregnancies, including live births and stillbirths, was divided into four groups: no child, one child, two children, and three or more children. Menopausal stages were classified based on the criteria of the Stages of Reproductive Aging Workshop + 10 as (1) premenopause; (2) early menopausal transition, defined as a persistent difference of ≥ 7 days in the length of consecutive cycles; (3) late menopausal transition, defined as amenorrhea of ≥ 60 days; and (4) postmenopause, defined as amenorrhea of ≥ 12 months^29,30^.

Participants wore a lightweight hospital gown and no shoes, and their height, weight, and body composition were measured by trained experts. Body mass index (BMI) was calculated as weight in kilograms divided by height in meters squared (kg/m^2^). Hypertension was defined as a systolic blood pressure (SBP) ≥ 130 mmHg, diastolic blood pressure (DBP) ≥ 80 mmHg^31^, or ongoing use of blood pressure-lowering drugs.

Blood samples were collected from the antecubital vein after at least 10 h of fasting. Fasting blood measurement included high-sensitivity C-reactive protein, glucose, hemoglobin A1c and insulin. Diabetes mellitus was defined as fasting glucose levels ≥ 126 mg/dL, glycated hemoglobin ≥ 6.5% (48 mmol/mol), or current use of insulin or glucose-lowering medication. Insulin resistance was evaluated by the homeostatic model assessment of insulin resistance (HOMA-IR) according to the following formula: HOMA-IR = fasting serum insulin (µIU/mL) × fasting serum glucose (mg/dL)/405 ^32^. Serum total cholesterol and TG concentrations were determined using an enzymatic colorimetric assay. HDL and LDL cholesterol levels were measured directly using a homogenous enzymatic colorimetric assay on a cobas 8000 c702 (Roche Diagnostics, Tokyo, Japan). The classification of lipoprotein lipid levels was based on the National Lipid Association Recommendations for Patient-Centered Management of Dyslipidemia^33^. We categorized total cholesterol levels into three groups: < 200 mg/dL, 200–239 mg/dL, and ≥ 240 mg/dL. Serum LDL cholesterol levels were divided into three groups: < 100 mg/dL, 100–129 mg/dL, and ≥ 130 mg/dL. HDL cholesterol concentrations were categorized as groups of low (< 50 mg/dL), borderline (50–59 mg/dL), and high (≥ 60 mg/dL); non-HDL-cholesterols were divided into three groups: normal (< 130 mg/dL), borderline (130–159 mg/dL), and high (≥ 160 mg/dL). As there were only 6% of women with serum triglyceride n with serum triglyg/dL 0 oups: ndL), and high ((130 0 odL)mg/dL) n < 100 mg/dL, 100–149 mg/dL, and ≥ 150 mg/dL.

The VMSs included hot flashes and night sweats. To determine the presence and degree of VMSs, the validated Korean version of the Menopause-Specific Quality of Life (MENQOL) questionnaire was administered at baseline and at each follow-up visit^34,35^. Study participants indicated whether they had experienced VMSs during the past month and described how bothersome the symptoms were on a seven-point Likert scale; from “not bothered at all” (0) to “extremely bothersome” (6)^34,36^. For the statistical analysis, the raw scores of VMSs intensity were recoded to an eight-point grading system including zero: the answer “No” was rescored as zero and “Yes, but not bothered at all” was converted to one. The increasing degree of VMSs severity ranging from 1 to 6 was rescored from 2 to 7. If the participant responded “No” to hot flashes or night sweats, we considered the participant as not having VMSs. Women who answered “Yes” and experienced hot flashes or night sweats were considered as having VMSs. We also considered groups who had ≥ 3 recoded points as having moderate/severe VMSs, and women with 1 or 2 points as having mild VMSs. Early-onset VMSs was defined as the occurrence of VMSs before menopause.

### Statistical analysis

Descriptive statistics were used to summarize the baseline characteristics of the study participants by early-onset VMSs. The differences in demographic and clinical characteristics at baseline between women with and without early-onset VMSs were analyzed using the t-test for continuous variables and Chi-squared test for categorical variables. The primary outcome was incident early-onset of VMSs. Based on self-reported surveys, we received participants’ information on VMSs and menopausal status at each visit. At baseline and during follow-up, VMSs, menopausal stages, and the last two menstruation dates were assessed based on the self-administered, structured questionnaire including the MENQOL. Each participant was followed from the time of the baseline visit to the time of first report of VMSs occurrence, the time of menopause, or the last time the questionnaire survey was completed, whichever came first. Each participant was followed from the time of the baseline visit to the time of VMSs occurrence, the time of menopause, or the last time the questionnaire survey was completed, whichever came first. If a woman was in the postmenopausal stage at the time of the first VMS report, follow-up ended at the preceding visit where MENQOL assessment was completed prior to menopause. Cox proportional hazards regression model was used to estimate hazard ratios (HRs) with 95% confidence intervals (CIs) for incident early-onset VMSs according to lipid profiles. For sensitivity analysis, we repeated the analyses using moderate/severe VMSs as an endpoint. Potential confounders included age, BMI, BP, diabetes, educational status, parity, physical activity, smoking status, and alcohol intake based on previous findings (also see Supplementary Figure 2)^37–41^. Confounding variables were chosen for inclusion in the multivariable models if they met the following criteria: (1) were associated with the outcome (incident early-onset VMS) and (2) were associated with the exposure (lipid profiles), but 3) were not intermediate variables in the causal pathway between the exposure (lipid profiles) and the outcome (incident VMS). For a linear trend test, the median value of each category was included as a continuous variable in the model. All statistical analyses were conducted using Stata version 17.0 (Stata Corp LP; College Station, TX, USA). Statistical significance was defined as a two-sided *P*-value of less than 0.05.

## Supplementary Information

Supplementary Information.

## Data Availability

There are no linked research data sets for this paper. Data will be made available on request sent by e-mail to Dr. Chang (yoosoo.chang@gmail.com).

## References

[1] Utian WH (2005). Psychosocial and socioeconomic burden of vasomotor symptoms in menopause: A comprehensive review. Health Qual. Life Outcomes.

[2] Gold EB (2006). Longitudinal analysis of the association between vasomotor symptoms and race/ethnicity across the menopausal transition: Study of women’s health across the nation. Am. J. Public Health.

[3] Sayan S, Pekin T, Yıldızhan B (2018). Relationship between vasomotor symptoms and metabolic syndrome in postmenopausal women. J. Int. Med. Res..

[4] Avis NE (2015). Duration of menopausal vasomotor symptoms over the menopause transition. JAMA Intern. Med..

[5] Freeman EW, Sammel MD, Lin H, Liu Z, Gracia CR (2011). Duration of menopausal hot flushes and associated risk factors. Obstet. Gynecol..

[6] Zhu D (2020). Vasomotor menopausal symptoms and risk of cardiovascular disease: A pooled analysis of six prospective studies. Am. J. Obstet. Gynecol..

[7] Gast GC (2008). Menopausal complaints are associated with cardiovascular risk factors. Hypertension.

[8] Thurston RC (2012). Vasomotor symptoms and lipid profiles in women transitioning through menopause. Obstet. Gynecol..

[9] Muka T (2016). Association of vasomotor and other menopausal symptoms with risk of cardiovascular disease: A systematic review and meta-analysis. PLoS ONE.

[10] Mešalić L, Tupković E, Kendić S, Balić D (2008). Correlation between hormonal and lipid status in women in menopause. Bosn. J. Basic Med. Sci..

[11] Moorthy K (2004). Estradiol and progesterone treatments change the lipid profile in naturally menopausal rats from different age groups. Biogerontology.

[12] Nasr A (2020). Vasomotor symptoms and lipids/lipoprotein subclass metrics in midlife women: Does level of endogenous estradiol matter? The SWAN HDL Ancillary Study. J. Clin. Lipidol..

[13] Randolph JF (2011). Change in follicle-stimulating hormone and estradiol across the menopausal transition: Effect of age at the final menstrual period. J. Clin. Endocrinol. Metab..

[14] Atsma F, Bartelink M-LE, Grobbee DE, van der Schouw YT (2006). Postmenopausal status and early menopause as independent risk factors for cardiovascular disease: A meta-analysis. Menopause.

[15] Gast GC, Samsioe GN, Grobbee DE, Nilsson PM, van der Schouw YT (2010). Vasomotor symptoms, estradiol levels and cardiovascular risk profile in women. Maturitas.

[16] Steinberg HO (1997). Endothelial dysfunction is associated with cholesterol levels in the high normal range in humans. Circulation.

[17] Hermida N, Balligand JL (2014). Low-density lipoprotein-cholesterol-induced endothelial dysfunction and oxidative stress: The role of statins. Antioxid Redox Signal.

[18] Tuomikoski P (2010). Biochemical markers for cardiovascular disease in recently postmenopausal women with or without hot flashes. Menopause.

[19] Thurston RC (2017). Menopausal symptoms and cardiovascular disease mortality in the Women's Ischemia Syndrome Evaluation (WISE). Menopause.

[20] Thurston RC (2016). Trajectories of vasomotor symptoms and carotid intima media thickness in the study of Women's Health Across the Nation. Stroke.

[21] Kroenke CH (2012). Effects of a dietary intervention and weight change on vasomotor symptoms in the Women’s Health Initiative. Menopause (New York, NY).

[22] Greendale GA, Gold EB (2005). Lifestyle factors: Are they related to vasomotor symptoms and do they modify the effectiveness or side effects of hormone therapy?. Am. J. Med..

[23] Lambert E (2013). Dyslipidemia is associated with sympathetic nervous activation and impaired endothelial function in young females. Am. J. Hypertens.

[24] Thurston RC (2018). Physiologically assessed hot flashes and endothelial function among midlife women. Menopause.

[25] Thurston RC, Christie IC, Matthews KA (2010). Hot flashes and cardiac vagal control: A link to cardiovascular risk?. Menopause.

[26] Matthews KA (2009). Are changes in cardiovascular disease risk factors in midlife women due to chronological aging or to the menopausal transition?. J. Am. Coll. Cardiol..

[27] Craig CL (2003). International physical activity questionnaire: 12-country reliability and validity. Med. Sci. Sports Exerc..

[28] Kim K (2019). Smoking and urinary cotinine levels are predictors of increased risk for gastric intestinal metaplasia. Cancer Res..

[29] Harlow SD (2012). Executive summary of the Stages of Reproductive Aging Workshop+ 10: Addressing the unfinished agenda of staging reproductive aging. J. Clin. Endocrinol. Metab..

[30] Choi Y (2015). Menopausal stages and serum lipid and lipoprotein abnormalities in middle-aged women. Maturitas.

[31] Whelton P (2017). ACC/AHA/AAPA/ABC/ACPM/AGS/APhA/ASH/ASPC/NMA/PCNA guideline for the prevention, detection, evaluation, and management of high blood pressure in adults: A report of the American College of Cardiology/American Heart Association Task Force on Clinical Practice Guidelines. Hypertension.

[32] Matthews DR (1985). Homeostasis model assessment: Insulin resistance and beta-cell function from fasting plasma glucose and insulin concentrations in man. Diabetologia.

[33] Jacobson TA (2015). National lipid association recommendations for patient-centered management of dyslipidemia: Part 1-full report. J. Clin. Lipidol..

[34] Park JH, Bae SH, Jung YM (2020). Validity and reliability of the Korean version of the menopause-specific quality of life. J. Korean Acad. Nurs.

[35] Sydora BC (2016). Use of the Menopause-Specific Quality of Life (MENQOL) questionnaire in research and clinical practice: A comprehensive scoping review. Menopause.

[36] Hilditch JR (1996). A menopause-specific quality of life questionnaire: Development and psychometric properties. Maturitas.

[37] Namgoung, S. *et al.* Metabolically healthy and unhealthy obesity and risk of vasomotor symptoms in premenopausal women: Cross‐sectional and cohort studies. *BJOG*. 10.1111/1471-0528.17224 (2022). 10.1111/1471-0528.17224 PMC9541406 35596933

[38] Shobeiri F, Jenabi E, Poorolajal J, Hazavehei SMM (2016). The association between body mass index and hot flash in midlife women: A meta-analysis. J. Menopausal Med..

[39] Herber-Gast G-CM, Mishra GD (2014). Early severe vasomotor menopausal symptoms are associated with diabetes. Menopause.

[40] Franco OH (2015). Vasomotor symptoms in women and cardiovascular risk markers: Systematic review and meta-analysis. Maturitas.

[41] Cortés YI (2020). Impact of nulliparity, hypertensive disorders of pregnancy, and gestational diabetes on vasomotor symptoms in midlife women. Menopause (New York, NY).

