# Supplementary material for: High low-density lipoprotein cholesterol level is associated with an increased risk of incident early-onset vasomotor symptoms
Source: Sci Rep. 2022 Aug 27;12:14652. doi: 10.1038/s41598-022-19028-4 (PMC9420127; doi:10.1038/s41598-022-19028-4)

| **Supplementary table 1.** Longitudinal association between lipid profiles and incidence of each VMSs component among premenopausal women free of VMSs at baseline* | | | | | | | |
| --- | --- | --- | --- | --- | --- | --- | --- |
| Lipid profiles | Person-years (PY) | Early-onset cases | Incidence rate | Age-adjusted | | Multivariable-adjusted | |
|  |  |  | (cases per 100 PY) | HR (95% CI) | | HR (95% CI) | |
| Symptom of Hot flashes (n=2655) | | | | | | | |
| Total cholesterol (mg/dL) |  |  |  |  |  |  |  |
| <200 | 7,533.38 | 675 | 9.0 | Reference | | Reference | |
| ≥200 | 4,367.46 | 396 | 9.1 | 0.99 | (0.87-1.12) | 0.96 | (0.85-1.09) |
| *P-value* |  |  |  | 0.823 | | 0.515 | |
| LDL cholesterol (mg/dL) |  |  |  |  |  |  |  |
| <100 | 3271.75 | 265 | 8.1 | Reference | | Reference | |
| 100-129 | 4,882.87 | 447 | 9.2 | 1.16 | (0.99-1.35) | 1.17 | (1.00-1.37) |
| ≥130 | 3,746.22 | 359 | 9.6 | 1.25 | (1.07-1.47) | 1.24 | (1.05-1.46) |
| *P for trend* |  |  |  | 0.007 | | 0.012 | |
| HDL cholesterol (mg/dL) |  |  |  |  |  |  |  |
| ≥50 | 10,445.07 | 925 | 8.9 | Reference | | Reference | |
| <50 | 1,455.77 | 146 | 10.0 | 1.07 | (0.90-1.28) | 1.03 | (0.86-1.24) |
| *P-value* |  |  |  | 0.428 | | 0.739 | |
| Triglyceride (mg/dL) |  |  |  |  |  |  |  |
| <150 | 11160.47 | 988 | 8.9 | Reference | | Reference | |
| ≥150 | 740.37 | 83 | 11.2 | 1.23 | (0.98-1.54) | 1.13 | (0.89-1.43_ |
| *P-value* |  |  |  | 0.070 | | 0.333 | |
| Symptom of Night sweats (n=2798) | | | | | | | |
| Total cholesterol (mg/dL) |  |  |  |  |  |  |  |
| <200 | 7,906.98 | 737 | 9.3 | Reference | | Reference | |
| ≥200 | 4,642.10 | 471 | 10.1 | 1.08 | (0.96-1.21) | 1.07 | (0.95-1.20) |
| *P-value* |  |  |  | 0.186 | | 0.271 | |
| LDL cholesterol (mg/dL) |  |  |  |  |  |  |  |
| <100 | 3398.58 | 291 | 8.6 | Reference | | Reference | |
| 100-129 | 5,147.00 | 505 | 9.8 | 1.18 | (1.02-1.36) | 1.19 | (1.03-1.38) |
| ≥130 | 4,003.50 | 412 | 10.3 | 1.28 | (1.10-1.49) | 1.27 | (1.09-1.49) |
| *P for trend* |  |  |  | 0.001 | | 0.003 | |
| HDL cholesterol (mg/dL) |  |  |  |  |  |  |  |
| ≥50 | 10,954.73 | 1045 | 9.5 | Reference | | Reference | |
| <50 | 1,594.35 | 163 | 10.2 | 0.96 | (0.81-1.14) | 0.93 | (0.78-1.10) |
| *P-value* |  |  |  | 0.646 | | 0.381 | |
| Triglyceride (mg/dL) |  |  |  |  |  |  |  |
| <150 | 11762.87 | 1113 | 9.5 | Reference | | Reference | |
| ≥150 | 786.21 | 95 | 12.1 | 1.26 | (1.02-1.56) | 1.17 | (0.94-1.46) |
| *P-value* |  |  |  | 0.031 | | 0.162 | |
| Abbreviations: CI, confidence interval; HR, hazard ratio; LDL, low-density lipoprotein; HDL, high-density lipoprotein; VMSs, vasomotor symptoms . | | | | | | | |
| *Parametric proportional hazard models were used. The multivariable model was adjusted for age, body mass index, systolic and diastolic blood pressure, educational level, parity, physical activity, smoking status, and alcohol intake. | | | | | | | |

| **Supplementary table 2.** Longitudinal association between lipid profiles and incidence of VMS among premenopausal women free of VMS at baseline (n = 2,542)^*^ | | | | | | | | | | | | | | | |
| --- | --- | --- | --- | --- | --- | --- | --- | --- | --- | --- | --- | --- | --- | --- | --- |
| Lipid profiles | Person-years (PY) | | | Early-onset VMS | | Incidence rate | | | Age-adjusted | | | Multivariable-adjusted | | | |
|  |  |  |  |  |  | (cases per 100 PY) | | | HR (95% CI) | | | HR (95% CI) | | | |
| Total cholesterol (mg/dL) |  | | |  | |  | | |  |  | |  | |  | |
| <200 | 7,089.01 | | | 779 | | 11.0 | | | Reference | | | Reference | | | |
| 200-<240 | 3,363.14 | | | 380 | | 11.3 | | | 1.03 | (0.91-1.16) | | 1.00 | | (0.88-1.13) | |
| ≥240 | 749.75 | | | 85 | | 11.3 | | | 0.96 | (0.77-1.20) | | 0.94 | | (0.75-1.18) | |
| *P for trend* |  | | |  | |  | | | 0.990 | | | 0.667 | | | |
| LDL-C (mg/dL) |  | | |  | |  | | |  |  | |  | |  | |
| <100 | 3096.89 | | | 311 | | 10.0 | | | Reference | | | Reference | | | |
| 100-<130 | 4,570.95 | | | 522 | | 11.4 | | | 1.18 | (1.02-1.36) | | 1.20 | | (1.04-1.38) | |
| ≥130 | 3,534.06 | | | 411 | | 11.6 | | | 1.23 | (1.06-1.42) | | 1.22 | | (1.05-1.42) | |
| *P for trend* |  | | |  | |  | | | 0.008 | | | 0.013 | | | |
| HDL-C (mg/dL) |  | | |  | |  | | |  |  | |  | |  | |
| <50 (abnormal) | 1371.9 | | | 161 | | 11.7 | | | 0.97 | (0.82-1.15) | | 0.93 | | (0.78-1.12) | |
| 50-<60 | 2,441.16 | | | 275 | | 11.3 | | | 0.97 | (0.84-1.11) | | 0.96 | | (0.83-1.10) | |
| ≥60 | 7,388.84 | | | 808 | | 10.9 | | | Reference | | | Reference | | | |
| *P for trend* |  | | |  | |  | | | 0.657 | | | 0.395 | | | |
| Non-HDL(mg/dL) |  | | |  | |  | | |  |  | |  | |  | |
| <130 | 6767.28 | | | 733 | | 10.8 | | | Reference | | | Reference | | | |
| 130-<160 | 3028.68 | | | 345 | | 11.4 | | | 1.00 | (0.88-1.13) | | 0.98 | | (0.86-1.12) | |
| ≥160 | 1405.94 | | | 166 | | 11.8 | | | 1.04 | (0.88-1.23) | | 1.00 | | (0.84-1.20) | |
| *P for trend* |  | | |  | |  | | | 0.728 | | | 0.931 | | | |
| Triglycerides (mg/dL) |  | | |  | |  | | |  | | |  | | | |
| <100 | 8605.29 | | | 925 | | 10.7 | | | Reference | | | Reference | | | |
| 100-<150 | 1921.46 | | | 231 | | 12.0 | | | 1.13 | (0.98-1.31) | | 1.09 | | (0.94-1.27) | |
| ≥150 | 675.15 | | | 88 | | 13.0 | | | 1.19 | (0.95-1.48) | | 1.06 | | (0.84-1.35) | |
| *P for trend* |  | | |  | |  | | | 0.036 | | | 0.34 | | | |
| Abbreviations: VMS, vasomotor symptoms; HR, hazard ratio; *CI*, confidence interval; LDL, low-density lipoprotein; HDL, high-density lipoprotein. | | | | | | | | | | | | | | | |
| *The multivariable model was adjusted for age, body mass index, systolic and diastolic blood pressure, diabetes, educational level, parity, physical activity, smoking status, and alcohol intake. | | | | | | | | | | | | | | | |
| **Supplementary table 3.** Longitudinal association between lipid profiles and incidence of moderate/severe VMS among premenopausal women free of VMS at baseline (n = 2,542)^*^ | | | | | | | | | | | | | | |  |
| Lipid profiles | | Person-years (PY) | Onset of severe VMS | | Incidence rate | | Age-adjusted | | | | Multivariable-adjusted* | | | |  |
|  |  |  |  |  | (cases per 100 PY) | | HR (95% CI) | | | | HR (95% CI) | | | |  |
| Total cholesterol (mg/dL) | |  |  | |  | |  |  | | |  | |  | |  |
| <200 | | 7,433.40 | 342 | | 4.6 | | Reference | | | | Reference | | | |  |
| 200-<240 | | 3,535.44 | 183 | | 5.2 | | 1.12 | (0.94-1.34) | | | 1.07 | | (0.89-1.29) | |  |
| ≥240 | | 781.69 | 44 | | 5.6 | | 1.13 | (0.82-1.54) | | | 1.07 | | (0.78-1.48) | |  |
| *P for trend* | |  |  | |  | | 0.210 | | | | 0.456 | | | |  |
| LDL-C (mg/dL) | |  |  | |  | |  |  | | |  | |  | |  |
| <100 | | 3244.97 | 121 | | 3.7 | | Reference | | | | Reference | | | |  |
| 100-<130 | | 4,793.93 | 246 | | 5.1 | | 1.37 | (1.10-1.70) | | | 1.36 | | (1.09-1.69) | |  |
| ≥130 | | 3,711.63 | 202 | | 5.4 | | 1.46 | (1.16-1.83) | | | 1.39 | | (1.10-1.76) | |  |
| *P for trend* | |  |  | |  | | 0.002 | | | | 0.009 | | | |  |
| HDL-C (mg/dL) | |  |  | |  | |  |  | | |  | |  | |  |
| <50 (abnormal) | | 1,443.65 | 82 | | 5.7 | | 1.06 | (0.83-1.25) | | | 0.96 | | (0.74-1.24) | |  |
| 50-<60 | | 2,555.09 | 129 | | 5.0 | | 1.02 | (0.84-1.25) | | | 0.98 | | (0.80-1.21) | |  |
| ≥60 | | 7751.79 | 358 | | 4.6 | | Reference | | | | Reference | | | |  |
| *P for trend* | |  |  | |  | | 0.626 | | | | 0.759 | | | |  |
| Non-HDL(mg/dL) | |  |  | |  | |  |  | | |  | |  | |  |
| <130 | | 7089.24 | 320 | | 4.5 | | Reference | | | | Reference | | | |  |
| 130-<160 | | 3192.52 | 163 | | 5.1 | | 1.04 | (0.86-1.25) | | | 0.98 | | (0.81-1.20) | |  |
| ≥160 | | 1468.77 | 86 | | 5.9 | | 1.22 | (0.96-1.54) | | | 1.14 | | (0.89-1.45) | |  |
| *P for trend* | |  |  | |  | | 0.144 | | | | 0.442 | | | |  |
| Triglycerides (mg/dL) | |  |  | |  | |  | | | |  | | | |  |
| <100 | | 9008.44 | 415 | | 4.6 | | Reference | | | | Reference | | | |  |
| 100-<150 | | 2032.7 | 110 | | 5.4 | | 1.15 | (0.93-1.41) | | | 1.04 | | (0.84-1.30) | |  |
| ≥150 | | 709.39 | 44 | | 6.2 | | 1.26 | (0.92-1.72) | | | 1.04 | | (0.74-1.46) | |  |
| *P for trend* | |  |  | |  | | 0.072 | | | | 0.708 | | | |  |
| Abbreviations: VMS, vasomotor symptoms; HR, hazard ratio; *CI*, confidence interval; LDL, low-density lipoprotein; HDL, high-density lipoprotein. | | | | | | | | | | | | | | |  |
| *The multivariable model was adjusted for age, body mass index, systolic and diastolic blood pressure, diabetes, educational level, parity, physical activity, smoking status, and alcohol intake. | | | | | | | | | | | | | | |  |

| 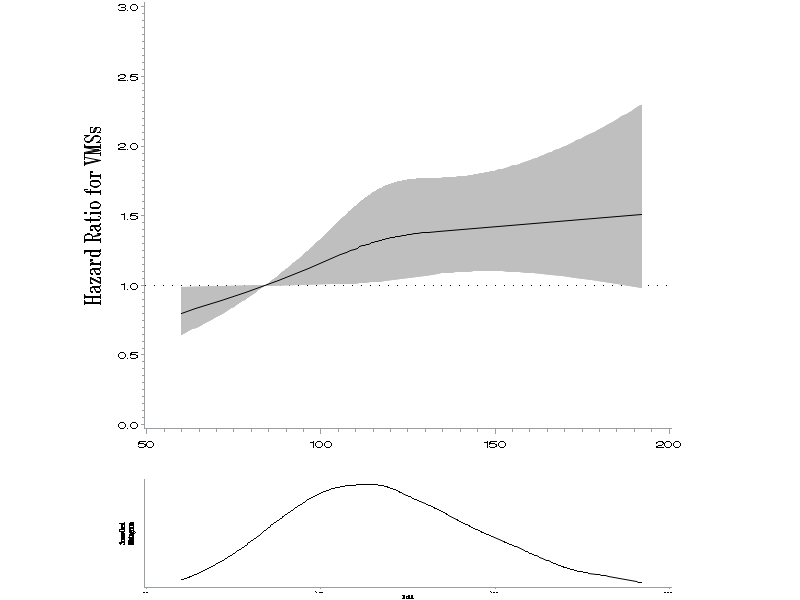 | 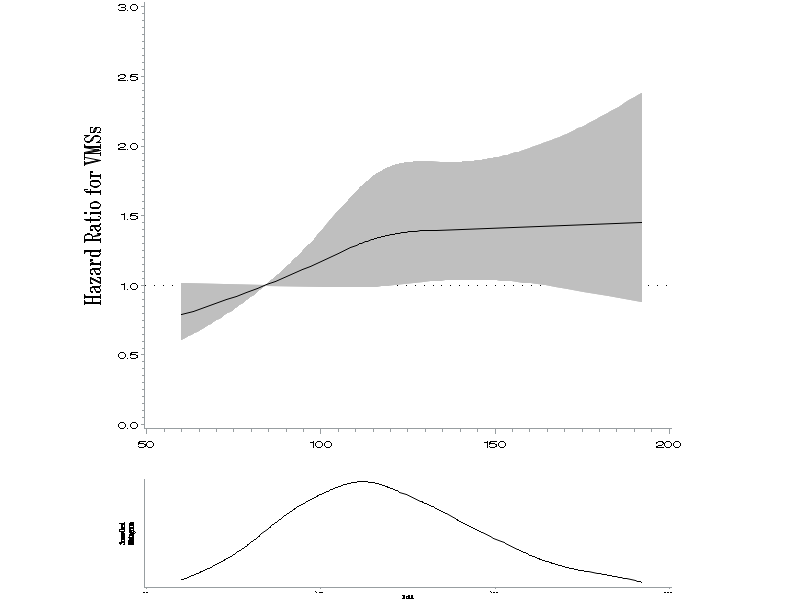 |
| --- | --- |
| (A) Unadjusted | (B) Adjusted |

**Supplementary Figure 1**. Restricted cubic spline curves of continuous hazard ratios for mild/moderate VMSs with LDL cholesterol levels in unadjusted (A) and adjusted (B) models

We examined the relationship between LDL cholesterol as a continuous variable and the risk of mild/moderate VMSs. For this analysis, we modeled LDL cholesterol as restricted cubic splines with knots at the 25^th^, 50^th^, and 75^th^ percentiles of the sample distribution to provide a flexible estimate of the concentration-response relationship between LDL cholesterol and VMS risk. With respect to the 10^th^ percentile of LDL cholesterol levels as the reference, the HRs of mild/moderate VMSs were increased as serum LDL cholesterol levels increased up to LDL levels <130 mg/dL. At higher LDL levels, the relationship between LDL and mild/moderate VMSs plateaued in both unadjusted and adjusted models.

**Supplementary Figure 2**. A directed acyclic graph of association between serum LDL cholesterol and early-onset VMS, using DAGitty.


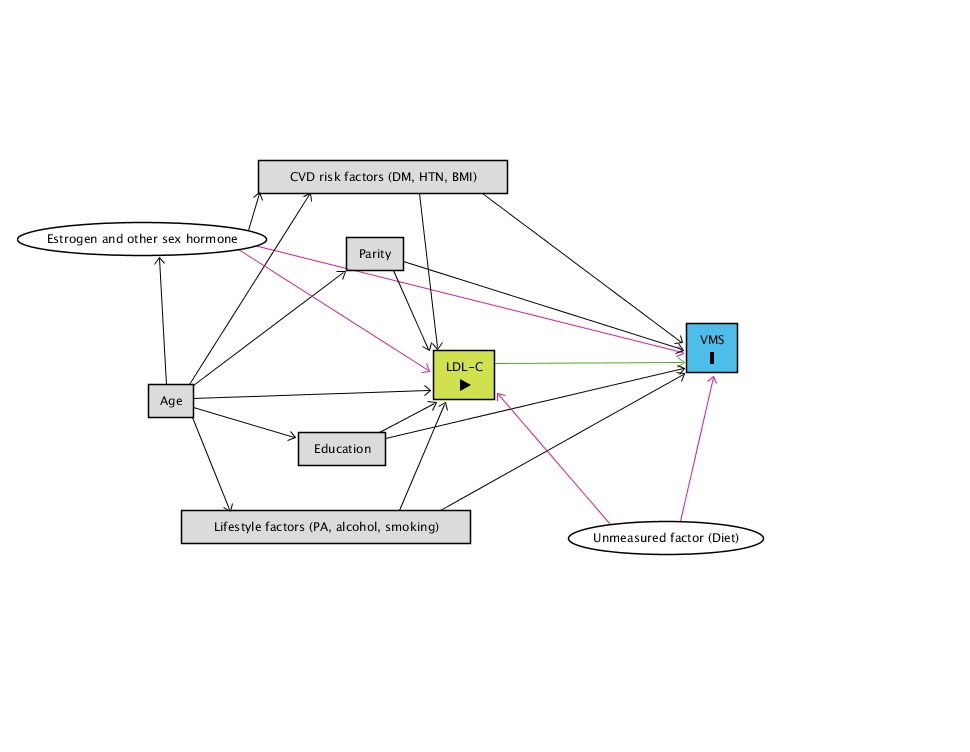

Supplement: Supplementary file 1 — Supplementary Information. [file 41598_2022_19028_MOESM1_ESM.docx]
